# MGFusion: a multimodal large language model-guided information perception for infrared and visible image fusion

**DOI:** 10.3389/fnbot.2024.1521603

**Published:** 2024-12-23

**Authors:** Zengyi Yang, Yunping Li, Xin Tang, MingHong Xie

**Affiliations:** ^1^Faculty of Information Engineering and Automation, Kunming University of Science and Technology, Kunming, Yunnan, China; ^2^Kunming Cigarette Factory, Hongyunhonghe Tobacco Group Company Limited, Kunming, Yunnan, China

**Keywords:** infrared and visible image fusion, CLIP, multimodal large language model, semantic information injection, image fusion

## Abstract

Existing image fusion methods primarily focus on complex network structure designs while neglecting the limitations of simple fusion strategies in complex scenarios. To address this issue, this study proposes a new method for infrared and visible image fusion based on a multimodal large language model. The method proposed in this paper fully considers the high demand for semantic information in enhancing image quality as well as the fusion strategies in complex scenes. We supplement the features in the fusion network with information from the multimodal large language model and construct a new fusion strategy. To achieve this goal, we design CLIP-driven Information Injection (CII) approach and CLIP-guided Feature Fusion (CFF) strategy. CII utilizes CLIP to extract robust image features rich in semantic information, which serve to supplement the information of infrared and visible features, thereby enhancing their representation capabilities for the scene. CFF further utilizes the robust image features extracted by CLIP to select and fuse the infrared and visible features after the injection of semantic information, addressing the challenges of image fusion in complex scenes. Compared to existing methods, the main advantage of the proposed method lies in leveraging the powerful semantic understanding capabilities of the multimodal large language model to supplement information for infrared and visible features, thus avoiding the need for complex network structure designs. Experimental results on multiple public datasets validate the effectiveness and superiority of the proposed method.

## 1 Introduction

In recent years, image fusion technology has garnered significant attention in the field of computer vision. Image fusion encompasses various types, including infrared and visible image fusion (Li and Wu, 2019), multi-exposure image fusion (Liu et al., 2022c; Li et al., 2024b; Tang et al., 2023a), multi-focus image fusion (Li et al., 2024a,c), medical image fusion (Liu et al., 2022e,d; Zhu et al., 2023, 2024), and remote sensing image fusion (Zhang Y. et al., 2024). Among these applications, infrared and visible image fusion technology stands out due to its wide range of applications. The infrared and visible image fusion technology aims to integrate a large amount of complementary information from both infrared and visible images to generate a single fused image, providing a more comprehensive description of the scene. Due to the differences in sensor imaging mechanisms, visible sensors can capture rich texture and color information (Zhang Y. et al., 2020; Xie et al., 2021). However, their performance is severely affected by lighting, weather, and smoke conditions. In contrast, infrared sensors can effectively capture thermal radiation information even under low-light and adverse weather conditions, highlighting targets such as people and vehicles in the images. By fusing infrared and visible images, it is possible to obtain information-rich scene images under all weather conditions. Consequently, this technology has found widespread applications in industrial control, autonomous driving, and aerospace fields.

In recent years, significant progress has been made in the research on infrared and visible image fusion to address various practical application challenges. These challenges primarily include inconsistencies in source image resolution (Li et al., 2021a; Ma et al., 2020; Xiao et al., 2022), unregistered source images (Xu et al., 2023; Li et al., 2023a,c; Wang et al., 2024), low-light environments (Chen et al., 2024; Tang et al., 2022, 2023b), extreme weather conditions (Yi et al., 2024; Li X. et al., 2024), and challenges in adapting to downstream task requirements (Zhang H. et al., 2024; Liu et al., 2023b; Liu Z. et al., 2023). In the effort to improve the quality of fused images, existing research primarily employs mainstream methods, including CNN feature interaction-based fusion methods (Li and Wu, 2019; Li et al., 2021b; Jian et al., 2021; Liu et al., 2022b, 2021; Li et al., 2023b; Yue et al., 2023), multiple feature extraction mechanisms-based fusion methods (Zhao et al., 2023; Li J. et al., 2021; Dong et al., 2024), and loss function-driven fusion methods (Liu et al., 2023a, 2022a; Zhou et al., 2023). These approaches aim to enhance the scene representation capability of multimodal features, thereby contributing to the overall quality of the fused images. Initially, researchers commonly designed feature extraction network structures based on convolutional neural networks (CNN), injecting more information into multimodal features through frequent information interactions to enhance the quality of fused images (Li and Wu, 2019; Li et al., 2023b; Yue et al., 2023). In these methods, many studies introduced skip connections (Jian et al., 2021), dense connections (Li and Wu, 2019), and nest connections (Li et al., 2021b) during feature extraction to enhance information exchange between features at different depths, thereby alleviating information loss caused by deeper networks. Additionally, some studies (Li et al., 2021a; Huang et al., 2022) employed convolutional kernels with varying dilation rates and sizes for feature extraction, allowing information from a larger receptive field to be aggregated into multimodal features. However, CNN have limitations in extracting rich global information, leading to constrained representation capability of the extracted features. Consequently, many studies have integrated advanced feature extraction methods with CNN to address these deficiencies. These approaches incorporate Transformers (Ma et al., 2022; Tang et al., 2023c), Generative Adversarial Networks (GAN; Ma et al., 2021; Zhang et al., 2021), and Mamba (Dong et al., 2024) into the feature extraction process to assist CNN in extracting more global information, thereby enhancing the quality of fused images.

However, the aforementioned methods often require researchers to have extensive design experience and significant manual resources. To address this issue, some studies have introduced carefully designed loss functions without the need for complex network structures. These loss functions impose constraints on feature extraction networks, encouraging the extracted features to contain more information. In representative works, loss functions based on contrastive learning (Liu et al., 2023a), loss functions focusing on salient targets (Liu et al., 2022a), and loss functions guided by semantic information (Zhou et al., 2023) have been introduced to enhance the quality of fused images. However, these methods need to consider the balance among numerous hyperparameters to better utilize the carefully designed loss functions. For example, the process of balancing hyperparameters within the new loss functions and between the new and existing loss functions can be lengthy and tedious. This parameter tuning often requires a significant amount of computational resources.To mitigate this, many researchers have attempted to introduce advanced ideas from other fields into infrared and visible image fusion, significantly reducing the workload of network structure design and parameter tuning. These approaches incorporate advanced concepts such as diffusion models (Yue et al., 2023) and low-rank sparse decomposition (Li et al., 2023b, 2020) to better decompose features from different modalities and accurately capture these features. However, diffusion models typically involve a large number of parameters and computational requirements, making them challenging to deploy on resource-constrained platforms. Additionally, low-rank sparse decomposition methods may lead to information loss during the extraction of low-rank and sparse features, thereby affecting fusion quality.

To address the shortcomings of existing methods, this paper reconsiders the strategies for enhancing image quality in infrared and visible image fusion. A careful analysis of the limitations of current approaches reveals that incorporating robust semantic information from outside the fusion network to supplement multimodal features can effectively alleviate unavoidable issues. In recent years, multimodal large language models have demonstrated strong semantic understanding and zero-shot learning capabilities through pre-training on large-scale multimodal datasets. Among them, CLIP stands out as a powerful model trained on extensive image-text data, possessing strong multimodal representation capabilities and excellent generalization performance. It can extract high-dimensional semantic representations from images, which are not only rich in semantic information but also exhibit strong robustness. These attributes make features extracted by models like CLIP particularly suitable for providing supplementary information to the features in the fusion network, thereby enhancing the quality of fused images. Therefore, this paper innovatively proposes a multimodal large language model-based framework for infrared and visible image fusion, which can achieve high-quality fused images without the need for complex network structures.

To enrich the semantic information of features in the fusion network, this paper proposes an information injection method based on CLIP (Radford et al., 2021). This method uses the multimodal features extracted by CLIP to supplement the features in the fusion network, significantly enriching the semantic information of the features to be fused and enhancing their robustness. Additionally, to address the challenges posed by simple fusion strategies, such as element-wise addition or channel concatenation, in complex fusion scenarios, this paper introduces a CLIP-guided feature fusion strategy. This strategy leverages CLIP's strong semantic understanding capabilities to select and fuse the features, meeting the need to improve the quality of fusion results in complex situations. The proposed method deeply integrates CLIP with the fusion network, providing information supplementation, feature selection, and feature fusion for the multimodal features in the original fusion network, thereby significantly improving the quality of the fused images. The main contributions of this paper and the advantages of the proposed method are highlighted in the following aspects:

(1) We propose a framework for infrared and visible image fusion based on multimodal large language models. This framework significantly enhances the quality of fused images while overcoming the shortcomings of existing methods, providing new insights for improving the quality of infrared and visible image fusion.

(2) We introduce multimodal large language model to supplement the features in the fusion network, enriching the semantic information of the features to be fused and enhancing their robustness. Additionally, we embed the multimodal large language model into the feature fusion process and propose a fusion strategy. This strategy uses the multimodal large language model for feature selection and fusion, effectively addressing complex fusion scenarios.

(3) We deploy this method on several publicly available infrared and visible image fusion datasets and conduct quantitative and qualitative comparisons to validate its fusion performance. The experimental results demonstrate that the proposed method significantly outperforms existing methods in both visual quality and objective evaluation metrics.

The remaining content of this paper is organized as follows: Section 2 reviews related work; Section 3 elaborates on the proposed method in detail; Section 4 presents the experimental results and their analysis; Section 5 summarizes the paper and draws some conclusions.

## 2 Related work

In the research of infrared and visible image fusion focused on enhancing image quality, existing methods can be broadly classified into the following categories based on their specific implementation approaches: CNN feature interaction-based fusion methods, multiple feature extraction mechanisms-based fusion methods, and loss function-driven fusion methods.

### 2.1 CNN feature interaction-based fusion methods

Fusion methods based on CNN feature interaction typically utilize convolutional neural networks (CNN) to construct feature extraction networks. They enrich feature representation through frequent information exchange between convolutional layers, thereby enhancing the quality of the fused images. In this category of methods, DenseFuse (Li and Wu, 2019) introduces dense connections in the feature encoder, promoting the fusion of multi-layer features through dense interactions between convolutional layers at different depths, ensuring that the output features contain as much rich information as possible from various layers. RFN-Nest (Li et al., 2021b) further fuses features of different depths within the encoder and inputs the multiple fused features into the decoder for deeper interaction and fusion. However, these methods do not adequately address the potential information loss that may occur between the encoder and decoder. To tackle this issue, SEDRFuse (Jian et al., 2021) introduces skip connections between the feature encoder and decoder, leveraging long-range information supplementation to reduce information loss during the forward propagation process.

Although the aforementioned methods enrich feature representation through frequent information exchange, they do not address the limitation of receptive fields in CNNs. To this end, MLFusion (Li et al., 2021a) is inspired by the human population Receptive Field (pRFs; Liu et al., 2018) and employs convolutional kernels of varying dilation rates and sizes for feature extraction, aggregating features from different receptive fields to obtain information from a larger receptive field. However, these methods overlook the shortcomings of CNNs in extracting global information, which limits the representational capacity of the extracted features.

### 2.2 Multiple feature extraction mechanisms-based fusion methods

Multiple feature extraction mechanisms-based fusion methods extract more comprehensive features by combining advanced feature extraction mechanisms with CNNs, thereby enhancing the quality of fused images. For example, SwinFusion (Ma et al., 2022) utilizes CNNs to extract basic features and further processes these features through Transformers to inject more global information. However, the extraction of global information in this method relies on the basic features extracted by CNNs, inevitably leading to the loss of some global information. To address this issue, CDDFuse (Zhao et al., 2023) employs Transformers and CNNs in parallel for feature extraction, merging the two to create features that contain both rich local and global information. In recent years, the Mamba model has gained widespread attention in the field of deep learning due to its advantages in efficiency, speed, scalability, and complexity management compared to Transformers. Consequently, Fusion-Mamba (Dong et al., 2024) introduces the Mamba model into the fusion framework, combining it with CNNs for feature extraction and fusion to further enhance the quality of fused images.

Moreover, many studies have incorporated adversarial learning mechanisms between the fusion results and source images into fusion methods, encouraging extracted features to contain richer information. For example, FusionGAN (Ma et al., 2019b) supervises the fusion results using a visible image's discriminator, prompting the fusion network to inject more edge detail information into the fused image. However, single-discriminator methods can lead to an imbalance in modal information, weakening the scene representation capability of the fusion results. To address this issue, DDcGAN (Ma et al., 2020) introduces a dual-modal discriminator into the fusion process, encouraging a more balanced injection of information from both infrared and visible images into the fusion results. Nevertheless, these methods often require researchers to possess extensive design experience and invest significant human resources.

Additionally, LRRNet (Li et al., 2023b) employs the concept of low-rank sparse decomposition, separating image features into low-rank and sparse components, and fuses these two parts separately to improve the quality of the reconstructed image. However, fusion methods based on low-rank sparse decomposition may lead to information loss during feature decomposition. resulting in suboptimal fusion outcomes. In recent years, diffusion models have achieved significant success in the field of image generation. Dif-Fusion (Yue et al., 2023) utilizes diffusion models for the fusion of infrared and visible images to achieve high-quality fusion results. However, the large number of parameters and computational demands of diffusion models limit their application on platforms with constrained storage and computational resources. In contrast to these methods, this paper introduces a multimodal large language model to inject robust semantic information into the features of the fusion network, enriching feature representation. To address the challenges of image fusion in complex scenarios, we have developed a novel fusion module based on the multimodal large language model, aiming to achieve high-fidelity fused images.

### 2.3 Loss function-driven fusion methods

Loss function-driven fusion methods constrain feature extraction networks through carefully designed loss functions, encouraging the extraction of more easily overlooked information to enhance the quality of fused images. For example, SDDGAN (Zhou et al., 2023) constructs a semantic-related loss function using semantic segmentation results, promoting the injection of more semantic information into the fused image. However, this method has limitations in enhancing information in the regions of salient objects. To address this issue, TarDAL (Liu et al., 2022a) introduces a loss function based on Saliency Degree Weight (SDW), focusing on enhancing the information of salient objects in the fused image. However, this method overly focuses on enhancing the information of the target objects, while the processing of background information is relatively weak. To counter this, CoCoNet (Liu et al., 2023a) incorporates contrastive learning into the fusion process, balancing the enhancement of both target and background information. In the regions of salient objects, the distance between the fused image and the infrared image is reduced, while the distance to the visible image is increased; conversely, in the background regions, the fused image is brought closer to the visible image, while the distance to the infrared image is increased. Nevertheless, such methods often require tedious and time-consuming parameter tuning to balance various hyperparameters, thereby fully leveraging the effectiveness of the loss function. In contrast to these methods, this paper introduces a multimodal large language model to inject robust semantic information into the features of the fusion network, enriching feature representation. To address the challenges of image fusion in complex scenarios, we have developed a novel fusion strategy based on the multimodal large language model, aiming to achieve high-fidelity fused images.

## 3 Proposed method

### 3.1 Overview

**Figure 1:**
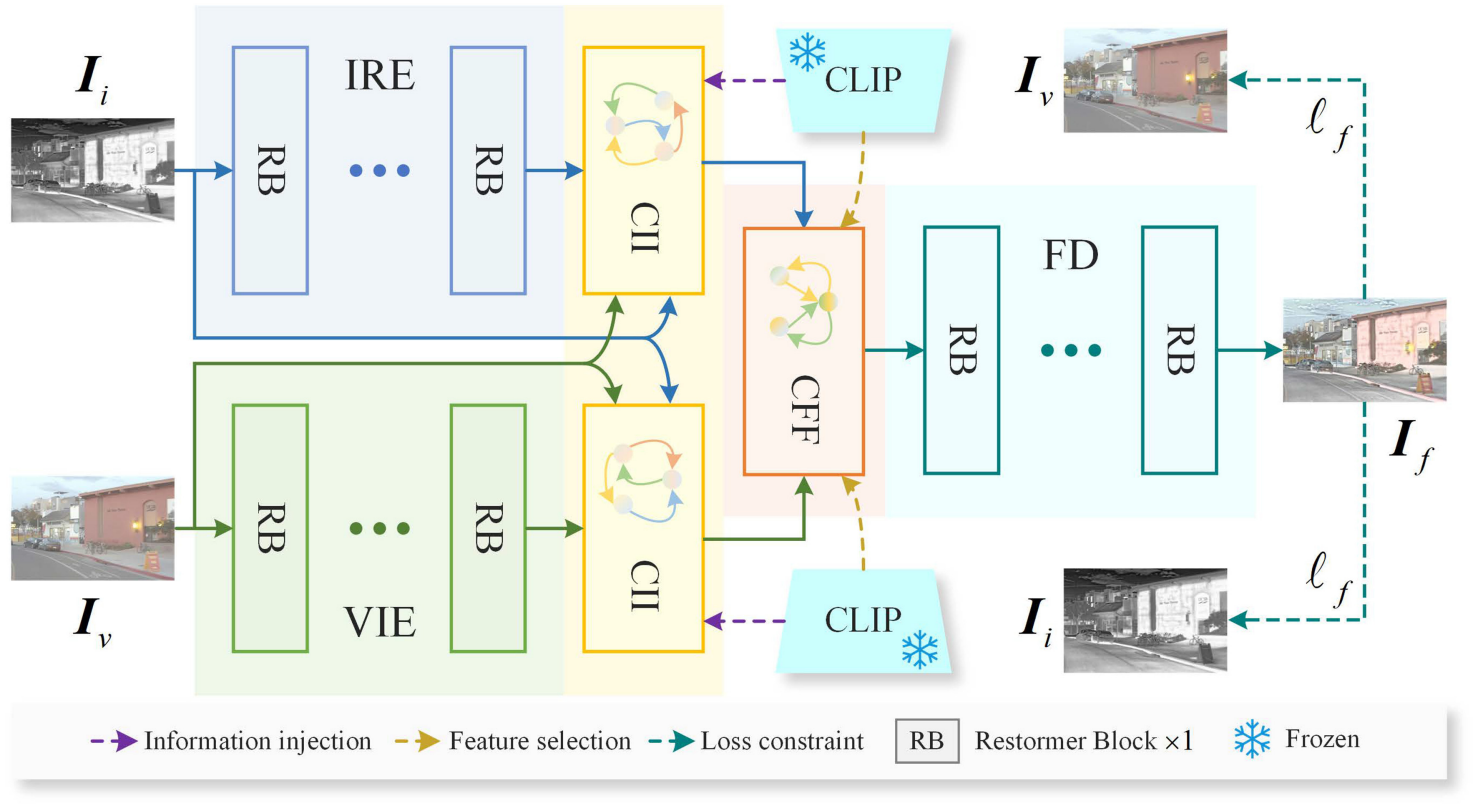
Overview of the proposed method. The IRE and VIE are employed to extract features from infrared and visible images, respectively. To enhance the features' ability to represent the scene, the extracted infrared and visible features are fed into the CII block, where CLIP is employed to inject rich semantic information into them. Following the semantic injection, the features are passed into the CFF block, which leverages CLIP to perform selection and fusion of the multimodal features. Finally, the fused features are input into the FD to reconstruct the fused image. Where, Restormer Block refers to a module proposed in Zamir et al. (2022).

As shown in Figure 1, the proposed method consists of five core components: the Infrared Feature Encoder (IRE), the Visible Feature Encoder (VIE), the CLIP-driven Information Injection (CII) block, the CLIP-guided Feature Fusion (CFF) block, and the Fusion Feature Decoder (FD). The IRE and VIE are designed to extract features from infrared images *I*_*i*_ and visible images *I*_*v*_, respectively. The CII block leverages CLIP to extract image features enriched with semantic information and injects this semantic content into the infrared and visible features, enhancing their ability to represent the scene. The CFF block further employs the robust features extracted by CLIP to select and fuse the features with injected semantic information, producing fused features. Finally, the FD decodes the fused features to reconstruct the fused image *I*_*f*_. In the following sections, we will provide a detailed explanation of each core component.

### 3.2 Feature extract and information injection

The network architectures of the IRE and VIE are identical, consisting primarily of two feature extraction layers followed by *N* Restormer Blocks (Zamir et al., 2022). Each feature extraction layer is composed of a convolutional layer with a kernel size of 3 × 3 and a stride of 1, stacked with a Batch Normalization layer and a LeakyReLU activation function layer. The infrared images $I_{i}\in {\mathbb{R}}^{H\times W\times 1}$ and visible images $I_{v}\in {\mathbb{R}}^{H\times W\times 3}$ are input into the IRE and VIE, respectively, to extract the infrared features $F_{i}\in {\mathbb{R}}^{H\times W\times C}$ and visible features $F_{v}\in {\mathbb{R}}^{H\times W\times C}$, where *H* and *W* represent the height and width of the source image, and *C* represents the number of feature channels.

**Figure 2:**
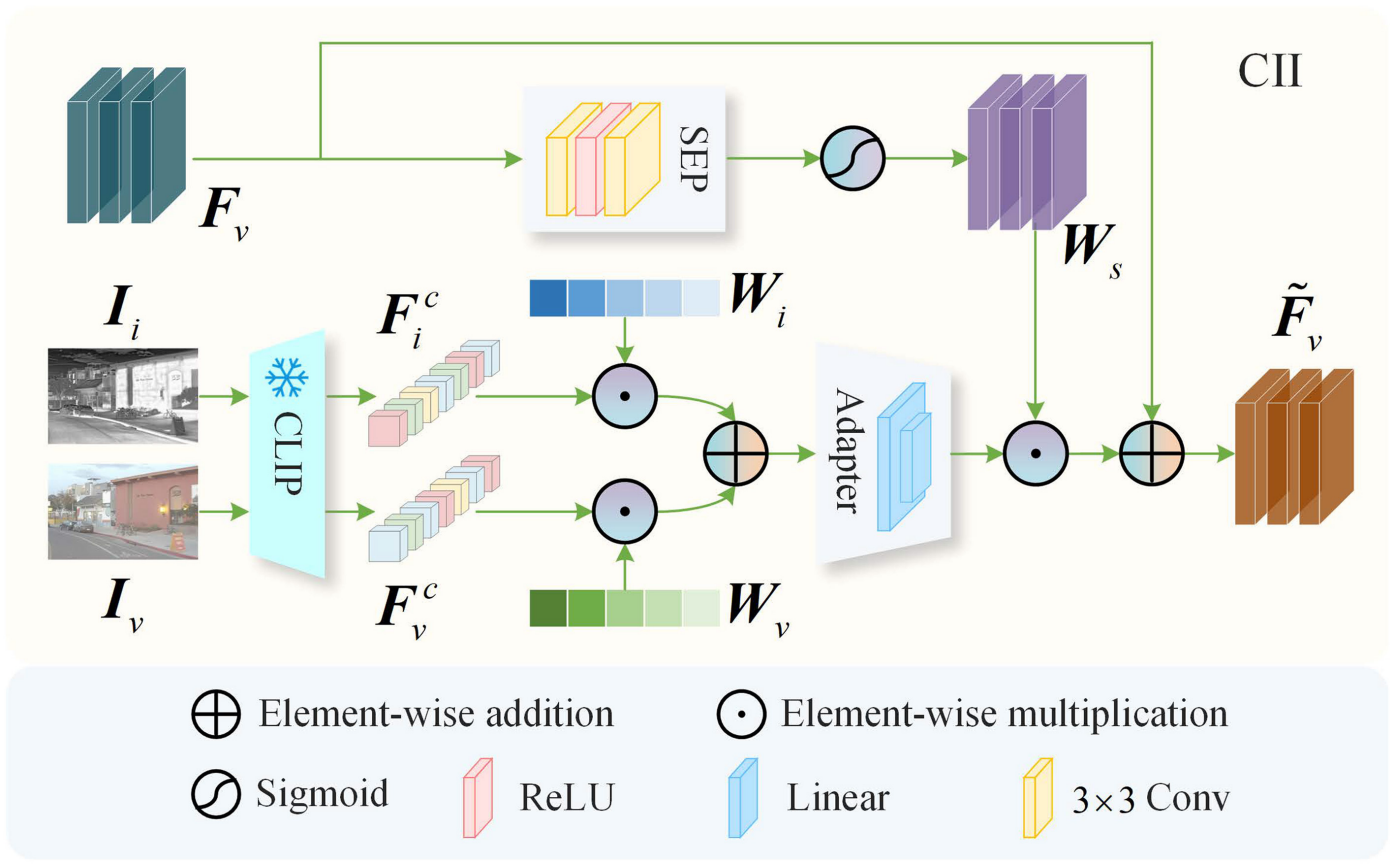
CLIP-driven information injection.

The infrared and visible features obtained through simple feature extraction often lack rich semantic information, making it challenging to achieve high-quality fusion results. Therefore, we directly utilize the pre-trained weights provided by the authors of CLIP, without any additional retraining, to leverage its powerful feature extraction capabilities. By injecting the rich semantic information extracted by CLIP into the infrared and visible features, we effectively enhance the quality of the fusion output. As illustrated in Figure 2, the CII block primarily consists of a frozen parameter pre-trained CLIP, an Adapter, and a Spatial Expansion Weight Prediction (SEP) block. The frozen pre-trained CLIP is utilized to extract image features rich in semantic content. The Adapter maps the integrated CLIP features into the same space as the infrared or visible features, unifying the number of channels across features to ensure that the features extracted by CLIP can be effectively utilized to enhance the quality of fused images. The SEP generates a weight based on the input features, which is used to expand the CLIP features to match the spatial dimensions of the input features. In terms of network architecture, the Adapter comprises two linear mapping layers, while the SEP consists of two convolutional layers with a kernel size of 3 × 3 and a stride of 1, and a single ReLU activation function layer. Taking the information injection process of the visible feature *F*_*v*_ as an example, we input the infrared image *I*_*i*_ and the visible image *I*_*v*_ into the frozen parameter image encoder of CLIP to obtain the CLIP features $F_{i}^{c}\in {\mathbb{R}}^{1\times 1\times E}$ and $F_{v}^{c}\in {\mathbb{R}}^{1\times 1\times E}$ for the infrared and visible images, respectively, where *E* represents the embedding dimension of the CLIP features. Considering that the features from different modalities contain a significant amount of complementary semantic information, we introduce a learnable weight $W_{i}\in {\mathbb{R}}^{1\times 1\times E}$ to integrate the semantic information from $F_{i}^{c}$ and $F_{v}^{c}$. The resulting output is then fed into the Adapter to ensure that the CLIP features are aligned in the same space as the visible feature *F*_*v*_:


(1) $$\begin{array}{l} F_{f}^{c}=\text{A}\left(F_{i}^{c}⊙W_{i}+F_{v}^{c}⊙W_{v}\right), \end{array}$$

where, $F_{f}^{c}\in {\mathbb{R}}^{1\times 1\times C}$ represents the integrated CLIP features, ⊙ denotes the Hadamard product, *W*_*v*_ = 1 − *W*_*i*_, and A(·) indicates the Adapter block. Simultaneously, the visible feature *F*_*v*_ is fed into the SEP, and the resulting output is passed through a Sigmoid activation function to obtain the weight *W*_*s*_ for expanding the CLIP feature space. To align $F_{f}^{c}\in {\mathbb{R}}^{1\times 1\times C}$ with $W_{s}\in {\mathbb{R}}^{H\times W\times C}$ in spatial dimensions, we utilize a broadcasting mechanism to achieve the spatial alignment. The broadcasting mechanism is a commonly used operation in the field of deep learning, which implicitly replicates the shape of smaller tensors to match that of larger tensors. The resulting output is then element-wise multiplied with *W*_*s*_ to obtain the semantic-rich feature $F_{s}^{c}\in {\mathbb{R}}^{H\times W\times C}$. Finally, we inject the semantic information into the visible feature *F*_*v*_ through an element-wise addition:


(2) $$\begin{array}{l} {\tilde{F}}_{v}=F_{v}+F_{s}^{c}, \end{array}$$

where, ${\tilde{F}}_{v}$ represents the visible feature enriched with semantic information. Similarly, we input the infrared image *I*_*i*_, the visible image *I*_*v*_, and the infrared feature *F*_*i*_ into the CII block to obtain the infrared feature ${\tilde{F}}_{i}$, which is enriched with semantic information.

### 3.3 Feature fusion and reconstruction

**Figure 3:**
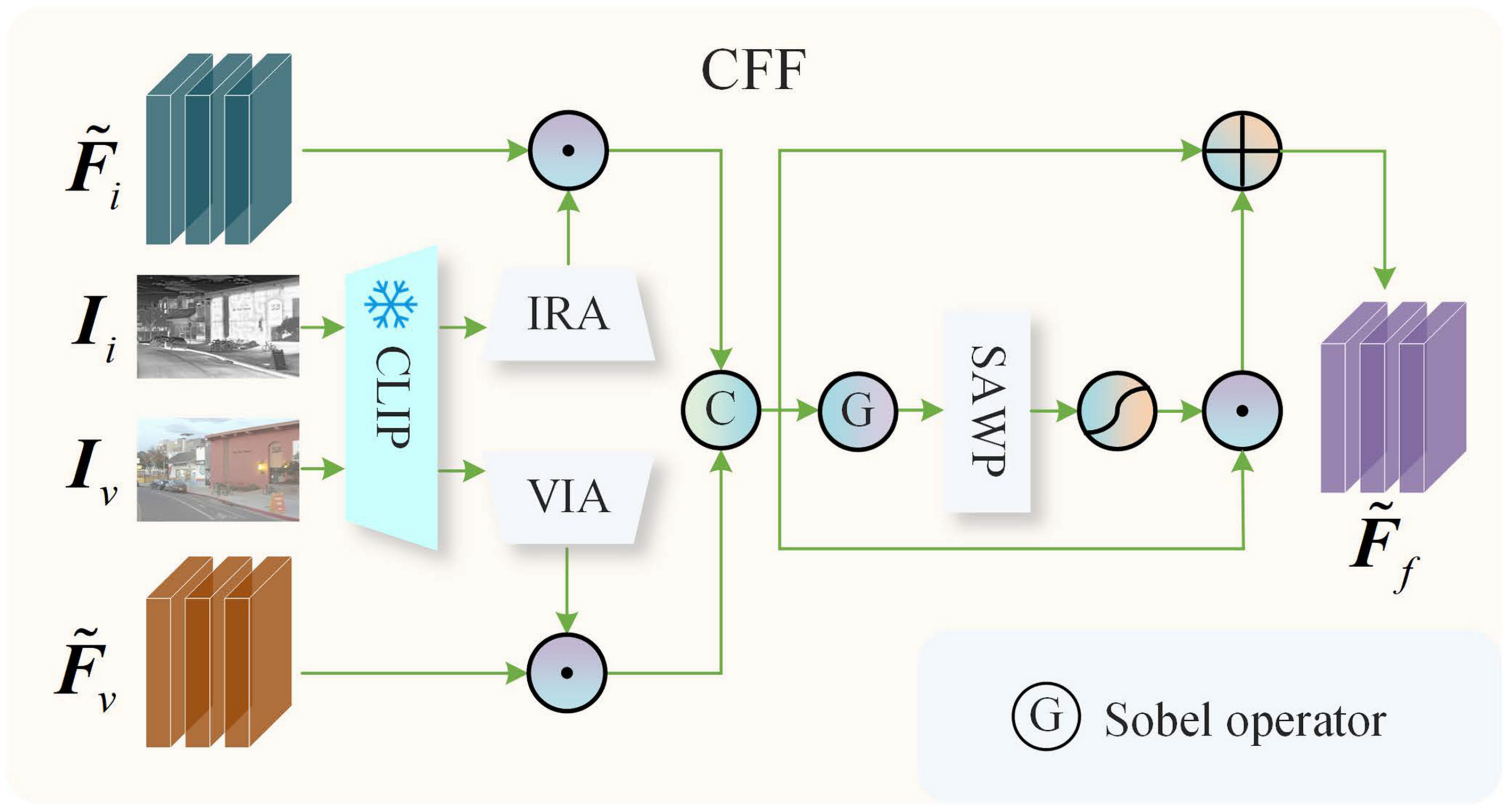
CLIP-guided feature fusion.

In existing fusion methods, fusion strategies typically involve element-wise addition or channel dimension concatenation, which often struggle to address image fusion in complex scenes, resulting in suboptimal fusion quality. To overcome these challenges, we leverage the robust feature representations extracted by the pre-trained CLIP to guide the fusion of infrared and visible features. As illustrated in Figure 3, the CFF block primarily comprises a frozen parameter pre-trained CLIP, an Infrared Adapter (IRA), a Visible Adapter (VIA), and a Spatial Attention Weight Prediction (SAWP) block. The IRA and VIA are responsible for generating attention weights that guide the fusion of infrared and visible features, respectively. The SAWP aggregates gradient information from the feature maps at the spatial level and generates weights to enhance texture details. In terms of network architecture, both the IRA and VIA are structured identically, consisting of two linear mapping layers. The SAWP is composed of two convolutional layers with a kernel size of 3 × 3 and a stride of 1, and a single ReLU activation function layer. In the CFF block, we input the infrared image *I*_*i*_ and the visible image *I*_*v*_ into the pre-trained CLIP image encoder, with the resulting features being fed into the IRA and VIA to obtain features $W_{i}^{f}\in {\mathbb{R}}^{1\times 1\times C}$ and $W_{v}^{f}\in {\mathbb{R}}^{1\times 1\times C}$, respectively. To guide the fusion of the infrared and visible features, we utilize a broadcasting mechanism to perform element-wise multiplication of $W_{i}^{f}$ and $W_{v}^{f}$ with ${\tilde{F}}_{i}$ and ${\tilde{F}}_{v}$, respectively, and concatenate the resulting outputs along the channel dimension:


(3) $$\begin{array}{l} F_{f}=\left[W_{i}^{f}⊙{\tilde{F}}_{i},W_{v}^{f}⊙{\tilde{F}}_{v}\right], \end{array}$$

where, $F_{f}\in {\mathbb{R}}^{H\times W\times C}$ represents the fused features, and [·] denotes the concatenation operation along the channel dimension.

To enhance the texture detail information within the fused features, we apply the Sobel operator for gradient extraction on *F*_*f*_, and the resulting gradient map is subsequently input into the SAWP and a Sigmoid activation function:


(4) $$\begin{array}{l} W_{g}\;\text{= Sigmoid(S}\left(\nabla F_{f}\right)\text{)}, \end{array}$$

where, *W*_*g*_ represents the spatial weights used to enhance texture details, while S(·) denotes the SAWP block, ∇ denotes the Sobel operator. We perform an element-wise multiplication of *W*_*g*_ and *F*_*f*_, and the resulting output is reinjected into *F*_*f*_ to enhance the texture detail information within *F*_*f*_:


(5) $$\begin{array}{l} {\tilde{F}}_{f}=F_{f}+W_{g}⊙F_{f}, \end{array}$$

where, ${\tilde{F}}_{f}$ represents the enhanced fused features. Finally, we input ${\tilde{F}}_{f}$ into the FD to reconstruct the fused image *I*_*f*_. The FD consists of *N* Restormer Blocks (Zamir et al., 2022), one feature extraction layer, and one image reconstruction layer. The image reconstruction layer is composed of a convolutional layer with a kernel size of 3 × 3 and a stride of 1, followed by a Tanh activation function layer.

To maximize the transfer of gradient information and pixel intensity information from the infrared and visible images to the fused image, we introduce a gradient loss ℓ_*g*_ and a pixel intensity loss ℓ_*i*_ to jointly construct the total fusion loss ℓ_*f*_:


(6) $$\begin{array}{l} \ell_{f}=\ell_{g}+\text{λ}\ell_{i}, \end{array}$$

where, λ represents the parameters used to balance the individual loss components. The gradient loss ℓ_*g*_:


(7) $$\begin{array}{l} \ell_{g}=\frac{1}{HW}||\nabla I_{f}-\max\left(\nabla I_{i},\nabla I_{v}\right){||}_{1}. \end{array}$$

And the pixel intensity loss ℓ_*i*_:


(8) $$\begin{array}{l} \ell_{i}=\frac{1}{HW}||I_{f}-\max\left(I_{i},I_{v}\right){||}_{1}, \end{array}$$

where, *H* and *W* represent the height and width of the fused image, respectively, ||·||_1_ denotes the *l*_1_-norm, and max(·) represents the element-wise maximum value.

## 4 Experiments

### 4.1 Datasets

We combined the RoadScene (Xu et al., 2022), M^3^FD (Liu et al., 2022a), MSRS (Tang et al., 2022), and LLVIP (Jia et al., 2021) datasets into a unified dataset and performed end-to-end training of the fusion network on this unified dataset. This unified dataset includes diverse scenes from both daytime and nighttime as well as infrared images from different spectral bands. Training the fusion network on this unified dataset significantly enhances its generalization ability when processing source images from varying scenes and spectral bands. Additionally, we validated the fusion performance of our method on five datasets: RoadScene, LLVIP, MSRS, M^3^FD, and TNO (Toet, 2017). Our experimental setup strictly follows the standard protocols in the domain. Specifically, we randomly selected 200, 201, 217, and 230 pairs of infrared and visible images from RoadScene, LLVIP, MSRS, and M^3^FD, respectively, as the training set. To enhance the diversity of the training samples, we applied various data augmentation techniques, including random flipping, random rotation, and random cropping (with a cropping size of 256 × 256). Furthermore, we randomly selected 20 pairs of infrared and visible images from each of the four datasets as the test set to evaluate the performance of the proposed method under supervised learning. To verify the generalization capability of the proposed method, we randomly selected 55 pairs of infrared and visible images from TNO as the test set and assessed the model's generalization performance on this dataset.

### 4.2 Implementation details

The method proposed in this paper use the Adam Optimizer (Kingma and Ba, 2015) to update the network parameters, with a batch size of 16 and a total of 100 training epochs. During training, a dynamic learning rate adjustment strategy is utilized: the learning rate gradually increases from an initial value of 1 × 10^−4^ to 1 × 10^−3^ over the first 20 epochs, and then decreases from 1 × 10^−3^ to 1 × 10^−4^ after the 20th epoch. Additionally, we set the hyperparameter λ to 0.2. This method is implemented using the PyTorch framework and trained on a single NVIDIA GeForce RTX 4090 GPU.

### 4.3 Evaluation metrics

To quantitatively compare the method proposed in this paper with existing methods, we adopted six widely used objective evaluation metrics in the field of image fusion: Gradient-based fusion performance (*Q*_*AB*/*F*_; Xydeas and Petrovic, 2000), Chen-Varshney metric (*Q*_*CV*_; Chen and Varshney, 2007; Liu Y. et al., 2024), Structural similarity index measure (*Q*_*SSIM*_; Wang et al., 2004), Average gradient (*Q*_*AG*_; Zhang X. et al., 2020), Visual information fidelity (*Q*_*VIF*_; Ma et al., 2019a), and Sum of correlation differences (*Q*_*SCD*_; Aslantas and Bendes, 2015). Among these metrics, *Q*_*AB*/*F*_ measures the retention of edge information from the source images in the fused image. A higher value indicates that the fused image contains richer edge information. *Q*_*CV*_ evaluates the quality of the fused image based on human visual perception, with smaller values indicating better perceptual quality. *Q*_*SSIM*_ assesses the similarity between the fused image and the source images in terms of brightness, contrast, and structure. A larger value suggests less information loss and lower distortion in the fused image. *Q*_*AG*_ quantifies the texture detail information in the fused image, with larger values indicating richer texture details. *Q*_*VIF*_ evaluates the shared information between the fused image and the source images based on human visual systems. A higher value indicates better visual fidelity of the fused image. *Q*_*SCD*_ uses the differential image between the source and fused images to assess the amount of information transfer. Larger values suggest smaller information differences between the fused and source images. Among these metrics, *Q*_*AB*/*F*_, *Q*_*SSIM*_, *Q*_*AG*_, *Q*_*VIF*_, and *Q*_*SCD*_ are positive indicators, meaning that larger values indicate better fusion performance of the compared methods. In contrast, *Q*_*CV*_ is a negative indicator, where smaller values represent better fusion performance of the compared methods.

### 4.4 Comparison experiments

To validate the superiority of the method proposed in this paper compared to existing SOTA fusion methods, we designed two experimental setups. In the first experiment, we compared our method with advanced fusion methods to highlight its advantages in visual quality and objective evaluation. The second experiment aimed to assess the generalization capability of our proposed method, where we conducted qualitative and quantitative comparisons on the untrained dataset TNO. Through these two experimental setups, we aim to provide a comprehensive and precise evaluation of the fusion performance of our proposed method.

#### 4.4.1 Comparison with state-of-the-art methods

**Figure 4:**
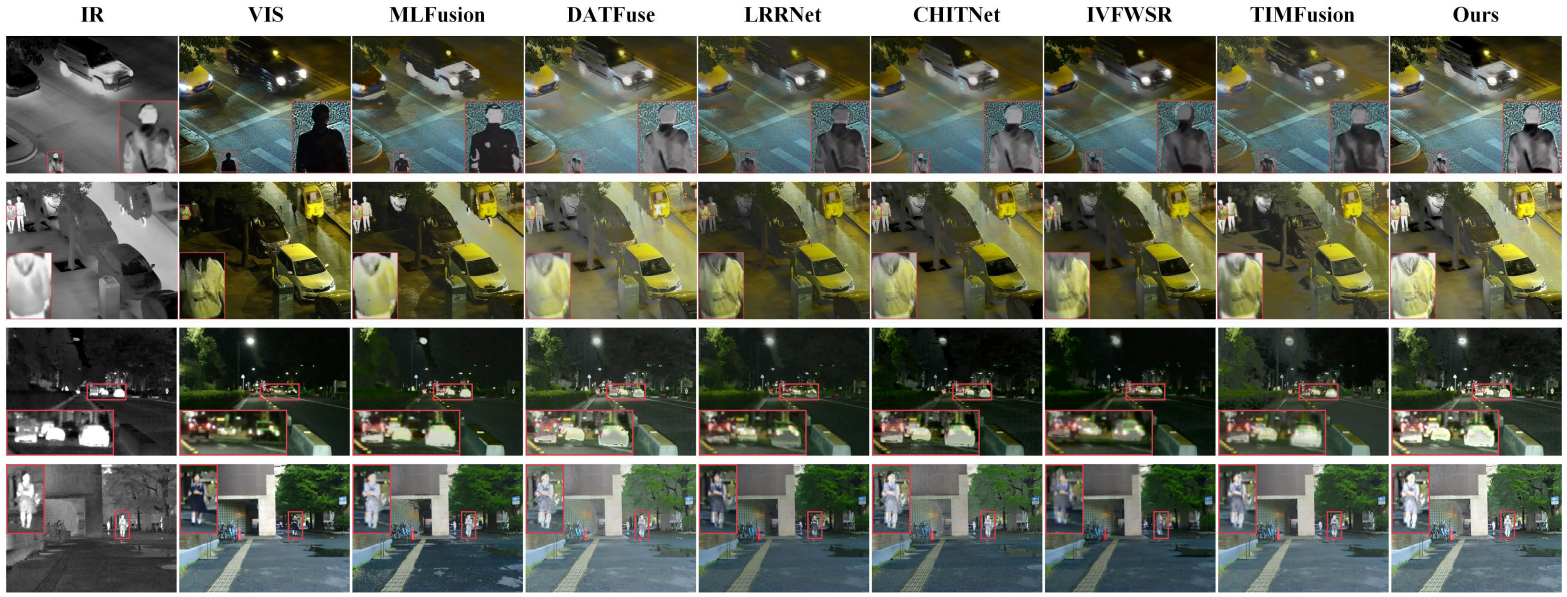
Compares the visual quality of the proposed method with SOTA fusion methods. The first and second columns show the infrared and visible images to be fused, respectively. The images in columns three through nine represent the fused results from various comparison methods. The first two rows of images are from the LLVIP dataset, while the last two rows are from the MSRS dataset.

**Figure 5:**
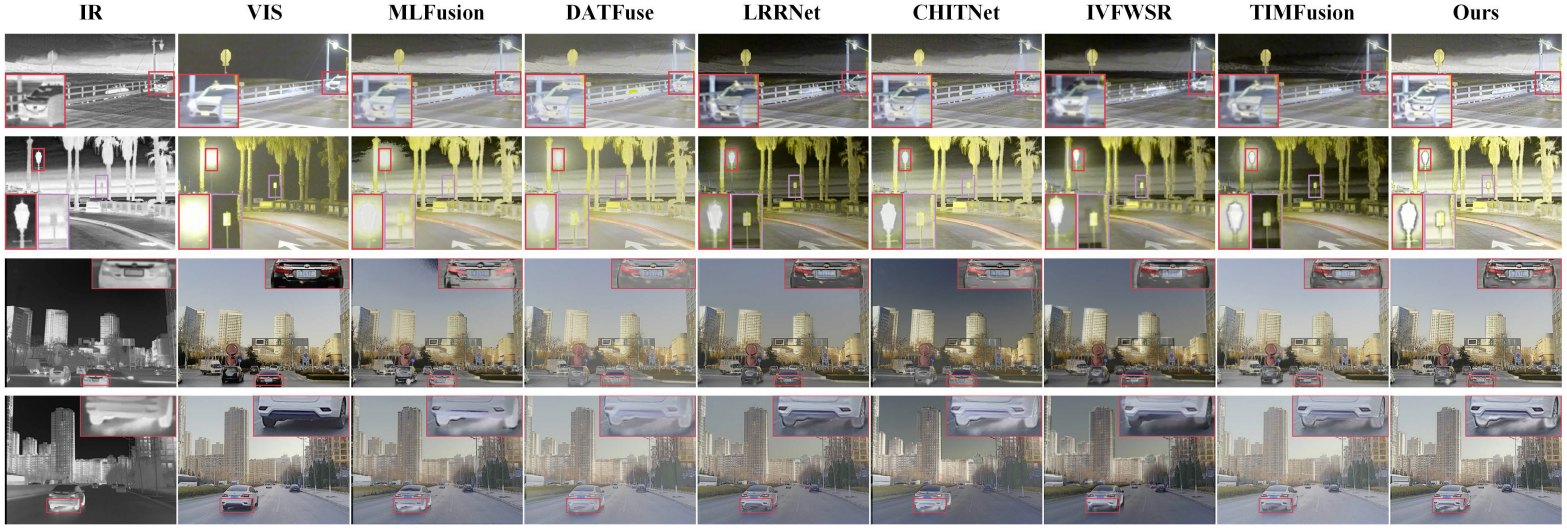
Compares the visual quality of the proposed method with SOTA fusion methods. The first and second columns show the infrared and visible images to be fused, respectively. The images in columns three through nine represent the fused results from various comparison methods. The first two rows of images are from the RoadScene dataset, while the last two rows are from the M^3^FD dataset.

We compared the method proposed in this paper with six advanced fusion methods: MLFusion (Li et al., 2021a), DATFuse (Tang et al., 2023d), LRRNet (Li et al., 2023b), CHITNet (Du, 2023), IVFWSR (Li et al., 2023a), and TIMFusion (Liu R. et al., 2024). The fusion results are shown in Figures 4, 5. The first two columns of Figures 4, 5 illustrate that there is substantial complementary information between infrared and visible images. Analysis of the overall brightness and contrast of the fused images indicates that our proposed method achieves higher contrast and brightness in both nighttime and daytime scenes, aligning better with human visual perception. This phenomenon is particularly evident in the second and third columns of Figure 4 and the second column of Figure 5. Compared to the other methods, the fused images generated by our proposed approach exhibit higher brightness and contrast for features such as sidewalks, distant vehicles, and clouds. To further emphasize the visual advantages of our method, we conducted zoom-in analysis on local regions. From these enlarged regions, it is evident that our proposed method achieves a better balance in preserving thermal radiation information and texture details for objects like pedestrians and vehicles. While significantly retaining thermal radiation information, the texture details of these objects remain clear. For example, in the zoomed-in region of the first column in Figure 5, some comparison methods show similar brightness for vehicles, but our proposed method displays more prominent and clearer texture details.

To further validate the superiority of the proposed method, we conducted a quantitative comparison of the fusion results using six commonly used objective evaluation metrics. The quantitative evaluation results are presented in Tables 1, 2. Analysis of Tables 1, 2 reveals that our proposed method outperforms most other methods in the average values of the six evaluation metrics. This advantage in fusion performance is particularly evident in metrics *Q*_*AB*/*F*_ and *Q*_*CV*_. In these two metrics, our method ranks first across the RoadScene, LLVIP, MSRS, and M^3^FD datasets and demonstrates significant superiority over other methods. In summary, our proposed method exhibits clear advantages in both visual quality and objective evaluation metrics compared to existing advanced methods.

**Table 1:** Quantitative evaluation results on the LLVIP dataset and the M^3^FD dataset.

**Methods**	**LLVIP**						**M** ^**3**^ **FD**					
	*Q*_*AB*/*F*_↑	*Q*_*CV*_↓	*Q*_*SSIM*_↑	*Q*_*AG*_↑	*Q*_*VIF*_↑	*Q*_*SCD*_↑	*Q*_*AB*/*F*_↑	*Q*_*CV*_↓	*Q*_*SSIM*_↑	*Q*_*AG*_↑	*Q*_*VIF*_↑	*Q*_*SCD*_↑
MLFusion	0.3275	553.87	1.2779	2.7637	0.2800	0.9936	0.4275	675.23	1.2943	4.5659	0.3719	1.2975
DATFuse	0.4691	413.56	1.2972	3.0195	0.3883	1.3473	0.4736	555.48	1.3046	4.1460	0.3314	1.3393
LRRNet	0.4206	572.32	1.3097	2.7998	0.2976	0.9901	0.5156	578.66	1.3634	4.5106	0.3166	1.3407
CHITNet	0.5252	775.84	1.3158	3.5493	0.3881	1.4497	0.4735	804.87	1.3692	4.9710	0.3294	1.4884
IVFWSR	0.2605	584.51	1.2757	2.3347	0.2070	1.2469	0.4472	718.98	1.2678	3.8479	0.2847	1.2975
TIMFusion	0.2895	886.35	1.1581	2.4373	0.3000	0.5524	0.5153	627.03	1.2875	4.3536	0.3190	1.1215
Ours	0.6950	272.64	1.3401	4.4622	0.4693	1.6128	0.6802	400.56	1.3089	6.0424	0.4180	1.5183

The top three results are highlighted using red, blue, and green.

**Table 2:** Quantitative evaluation results on the MSRS dataset and the RoadScene dataset.

**Methods**	**MSRS**						**RoadScene**					
	*Q*_*AB*/*F*_↑	*Q*_*CV*_↓	*Q*_*SSIM*_↑	*Q*_*AG*_↑	*Q*_*VIF*_↑	*Q*_*SCD*_↑	*Q*_*AB*/*F*_↑	*Q*_*CV*_↓	*Q*_*SSIM*_↑	*Q*_*AG*_↑	*Q*_*VIF*_↑	*Q*_*SCD*_↑
MLFusion	0.2825	798.82	1.3674	2.4207	0.2111	1.1943	0.4581	542.13	1.3629	4.0150	0.3936	1.3728
DATFuse	0.6299	404.84	1.2680	3.4412	0.4124	1.5900	0.4920	489.05	1.3506	4.2706	0.3523	1.3485
LRRNet	0.4241	677.40	1.2601	2.5557	0.2849	1.0710	0.3872	655.44	1.1970	5.1535	0.3458	0.9411
CHITNet	0.4748	783.83	1.2482	3.3012	0.3569	1.5300	0.4906	881.26	1.4049	5.1939	0.3452	1.5075
IVFWSR	0.3527	748.67	1.3172	2.1713	0.2462	1.2424	0.3219	1088.85	1.0306	4.0258	0.2242	1.0511
TIMFusion	0.3914	1132.22	1.1094	2.5886	0.3085	1.1499	0.3730	734.92	1.1951	4.4605	0.3914	1.0018
Ours	0.6666	327.47	1.3823	3.5313	0.4601	1.7657	0.5911	465.75	1.3325	5.8211	0.3731	1.5180

The top three results are highlighted using red, blue, and green.

#### 4.4.2 Verification of generalization ability

**Figure 6:**
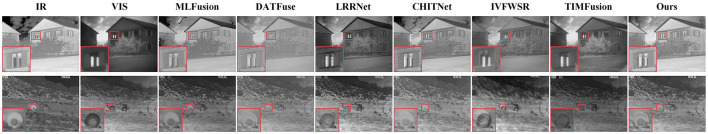
Compares the visual quality on the TNO dataset. The first and second columns show the infrared and visible images to be fused, respectively. The images in columns three through nine represent the fused results from various comparison methods.

We conducted qualitative and quantitative experiments on the untrained dataset TNO to verify the generalization capability of the proposed method. Specifically, we compared our proposed method with MLFusion, DATFuse, LRRNet, CHITNet, IVFWSR, and TIMFusion on the TNO dataset. The fusion results are presented in Figure 6. On the TNO dataset, the fused images generated by our proposed method maintain good brightness and contrast overall. As shown in the first row of Figure 6, the brightness and contrast of the building window areas surpass those of the other methods, allowing observers to quickly locate the position of the windows. Another advantage in visual quality lies in the preservation of texture details in local regions. For example, in the zoomed-in area of the second row in Figure 6, our proposed method retains better detail of the vehicle contours, providing a more accurate reflection of the vehicle's condition. In contrast, the fused images generated by other methods fail to comprehensively display the details of the vehicle wheels, lacking a good balance between brightness and texture, which hinders observers from quickly and accurately assessing the vehicle's status. As shown in Table 3, we conducted a quantitative assessment of the generalization capability of our proposed method. The results indicate that our proposed method achieves optimal or near-optimal levels across all metrics, demonstrating its superiority over other comparison methods. In summary, the results of both qualitative and quantitative comparisons indicate that our proposed method exhibits strong generalization capability.

**Table 3:** Quantitative evaluation results on the TNO dataset.

**Methods**	**TNO**					
	*Q*_*AB*/*F*_↑	*Q*_*CV*_↓	*Q*_*SSIM*_↑	*Q*_*AG*_↑	*Q*_*VIF*_↑	*Q*_*SCD*_↑
MLFusion	0.3909	379.92	1.3559	3.5907	0.4040	1.3990
DATFuse	0.4946	437.10	1.3693	3.7790	0.3769	1.3508
LRRNet	0.3588	834.27	1.3107	4.2212	0.4083	1.3431
CHITNet	0.4393	387.36	1.3804	5.0692	0.4192	1.6149
IVFWSR	0.3299	1381.49	1.2305	3.1430	0.2946	1.3100
TIMFusion	0.3915	695.57	1.2797	3.4787	0.4553	1.0899
Ours	0.5654	268.83	1.3921	4.4210	0.4352	1.5644

The top three results are highlighted using red, blue, and green.

### 4.5 Ablation study

The method proposed in this paper mainly consists of two core components: CLIP-driven Information Injection (CII) and CLIP-guided Feature Fusion (CFF). To validate the effectiveness of these two components, we conducted a series of ablation experiments on the MSRS dataset and performed qualitative and quantitative analyzes on the test set.

#### 4.5.1 Effectiveness of CII

**Figure 7:**
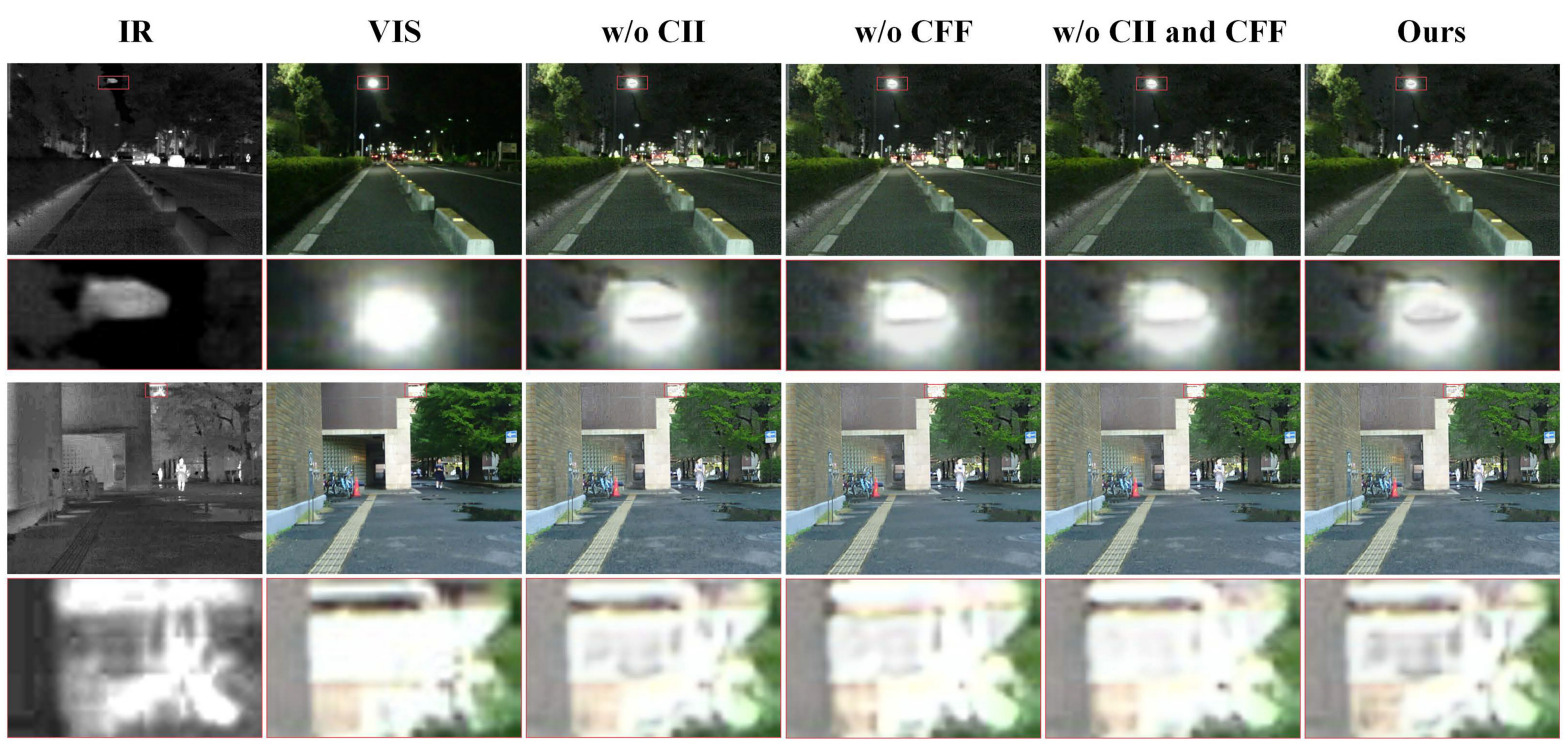
Qualitative results of the ablation study. The scene on the left half of the image is derived from the “00927N” image in the MSRS dataset, while the scene on the right half comes from the “00427D” image in the MSRS dataset.

In the method we propose, CII is a key component. It utilizes image features extracted by CLIP to inject semantic information into infrared and visible features, thereby enhancing the features' representation capability for the scene. To evaluate the effectiveness of CII, we conducted experiments by removing CII from the fusion framework and directly inputting the infrared and visible features obtained from IRE/VIE into CFF for subsequent processing. The results of the ablation experiments, shown in Figure 7, indicate that the model lacking CII exhibits a significant deficiency in detail information when fusing features of streetlights and distant buildings. This suggests that the absence of additional semantic information injection leads to a decline in the quality of the fused images. In contrast, our method, with semantic information injection, demonstrates richer texture details and better image quality. To further assess the impact of CII on image quality enhancement, we conducted quantitative comparisons across six evaluation metrics, as shown in Table 4. Analysis of Table 4 reveals that our method outperforms the model lacking CII on most evaluation metrics, further validating the effectiveness of CII.

**Table 4:** Quantitative evaluation results of the ablation experiments on MSRS dataset.

**Methods**	***Q*_*AB*/*F*_↑**	***Q*_*CV*_↓**	***Q*_*SSIM*_↑**	***Q*_*AG*_↑**	***Q*_*VIF*_↑**	***Q*_*SCD*_↑**
w/o CII	0.6503	343.19	1.3730	3.5350	0.4456	1.7805
w/o CFF	0.6260	351.21	1.3659	3.5073	0.4389	1.7624
w/o CII and CFF	0.6228	339.40	1.3806	3.4875	0.4462	1.7938
Ours	0.6666	327.47	1.3823	3.5313	0.4601	1.7657

The top result is highlighted in red.

#### 4.5.2 Effectiveness of CFF

In the method we propose, CFF is a key component. It constructs a fusion strategy based on CLIP and a multimodal large language model for feature selection and fusion, addressing image fusion in complex scenes. To evaluate the effectiveness of CFF, we removed it from the fusion framework and directly concatenated the infrared and visible light features output by CII along the channel dimension before inputting them into FD for image reconstruction. From the zoomed-in areas in Figure 7, it can be observed that the model lacking CFF experiences significant information loss when fusing features of illuminated streetlights and overexposed buildings, making it difficult to retain information from the source images. In contrast, our method effectively aggregates information from source images in such complex scenes, producing higher-quality fusion results. According to the quantitative comparison results in Table 4, the model without CFF is inferior to the complete model in the average values of all evaluation metrics. Combining both quantitative and qualitative comparisons, CFF plays an important role in image fusion for complex scenes.

## 5 Conclusion

This paper investigates the enhancement of image quality in infrared and visible image fusion and proposes a novel fusion method. To address the limitations of existing methods that rely on complex network architectures for improving image quality and to tackle the challenges of image fusion in complex scenarios, we introduce a multimodal large language model-driven approach for infrared and visible light image fusion. This method utilizes robust image features rich in semantic information extracted by CLIP to supplement the infrared and visible features, thereby meeting the high demand for semantic information in enhancing image quality. Furthermore, to address the complexities of fusion scenarios, we leverage CLIP's powerful semantic understanding capabilities to select and fuse infrared and visible features. Extensive qualitative and quantitative experiments demonstrate a significant improvement in the effectiveness and superiority of our proposed method compared to existing approaches. Our method is primarily designed for the fusion of infrared and visible images. When directly applied to other image fusion tasks, such as multi-focus image fusion, multi-exposure image fusion, or medical image fusion, its performance may decline. To address this issue, task-specific loss functions need to be introduced, and the network needs to be retrained to maintain satisfactory fusion performance. In light of the limitations of the proposed method, future research will focus on expanding the application of multimodal large language models to other image fusion tasks. Additionally, we will conduct an in-depth exploration of the commonalities among multimodal large language models and incorporate more diverse types of these models to further enhance the quality of fused images.

## Data Availability

Publicly available datasets were analyzed in this study. The datasets for this study can be found in the RoadScene dataset: https://github.com/hanna-xu/RoadScene, the MSRS dataset: https://github.com/Linfeng-Tang/MSRS, the LLVIP dataset: https://github.com/bupt-ai-cz/LLVIP, the M3FD dataset: https://github.com/dlut-dimt/TarDAL, and the TNO dataset: https://figshare.com/articles/dataset/TNO Image Fusion Dataset/1008029.
